# Activated protein C in septic shock: a propensity-matched analysis

**DOI:** 10.1186/cc10089

**Published:** 2011-03-08

**Authors:** Farid Sadaka, Jacklyn O'Brien, Matthew Migneron, Julie Stortz, Alexander Vanston, Robert W Taylor

**Affiliations:** 1 St. John's Mercy Medical Center/St Louis University, 621 S New Ballas Rd., Suite 4006B, St. Louis, MO 63141, USA

## Abstract

**Introduction:**

The use of human recombinant activated protein C (rhAPC) for the treatment of severe sepsis remains controversial despite multiple reported trials. The efficacy of rhAPC remains a matter of dispute. We hypothesized that patients with septic shock who were treated with rhAPC had an improved in-hospital mortality compared to patients with septic shock with similar acuity who did not receive rhAPC.

**Methods:**

This retrospective cohort study was completed at a large university-affiliated hospital. All patients with septic shock admitted to a 50-bed ICU between July 2003 and February 2009 were included. Patients were treated according to sepsis management guidelines.

**Results:**

A total of 563 septic shock patients were included (110 received rhAPC and 453 did not). Treated and untreated groups were matched in patient characteristics, comorbidities, and physiologic variables in a 1:1 propensity-matched analysis (108 received rhAPC, 108 did not). Mean Acute Physiology And Chronic Health Evaluation II (APACHE II) scores were 24.5 for the matched treated and 23.9 for the matched untreated group (*P *= 0.54). Receipt of rhAPC was associated with reduced in-hospital mortality (35.2% vs. 53.8%, *P *= 0.005), similar mean days on vasopressors (2 vs. 2, *P *= 0.90), similar mean days on mechanical ventilation (9 vs. 8.7, *P *= 0.80), similar mean length of ICU stay in days (11.0 vs. 11.3, *P *= 0.90), and similar mean length of hospital stay in days (19.5 vs 27, *P *= 0.11). No patients in either group had intracranial bleeding; differences in gastrointestinal bleeding and transfusion requirements were not statistically significant.

**Conclusions:**

Patients in our institution with septic shock who were treated with rhAPC had a reduced in-hospital mortality compared with patients with septic shock with similar acuity who were not treated with rhAPC. In addition, time on mechanical ventilation, time on vasopressors, lengths of stay and bleeding complications did not differ between the groups.

## Introduction

In the United States alone, approximately 750,000 cases of sepsis occur each year, of which at least 225,000 are fatal. One study evaluating the epidemiology of sepsis between 1979 and 2000 showed an 8.7% increase in the annual incidence of sepsis. The cost of management of a septic patient is approximately $50,000 amounting to annual costs estimated at $17 billion. Sepsis is the second leading cause of death in noncoronary Intensive Care Units (ICUs), and the 10^th ^leading cause of death overall. Organ failure occurs in 33.6% of patients with sepsis. Severe sepsis carries an estimated 30 to 50% mortality. Seventy percent of patients with three or more organ failures die. Those who survive severe sepsis have a lower quality of life compared to the age- and gender-adjusted general population, as much as 1.5 years later [1-7].

Sepsis is a complex syndrome that remains incompletely understood. It has proven very difficult to tailor new therapies for severe sepsis. One such potential therapy is Recombinant Human Activated Protein C (rhAPC). The use of rhAPC for the treatment of severe sepsis remains controversial despite multiple reported trials [8-13]. The Prospective Recombinant Human Activated Protein C Worldwide Evaluation in Severe Sepsis (PROWESS) trial reported a 6% absolute reduction in mortality together with a 1.5% absolute increase in the risk of serious bleeding in patients receiving rhAPC compared to those receiving placebo [8]. In a subgroup analysis of the PROWESS trial, patients with higher risk of death had a larger drop in hospital mortality compared to patients with a lower risk of death. In patients with Acute Physiologic and Chronic Health Evaluation II (APACHE II) score >25, hospital mortality dropped from 47.9% to 36.7% (*P *= 0.002) with rhAPC treatment [9]. In patients with three, four, or five organ failures, hospital mortality dropped from 37.0% to 30.5%, 50.1% to 44.9%, and 63.3% to 32.5%, respectively [9]. In contrast, a subsequent trial suggested that patients with lower mortality risk did not benefit [10]. Widespread clinical use of rhAPC has likely also been limited by its cost estimated at $7,500 to $9,000 per course of therapy [14-16].

Two large multicenter trials evaluating the effect of rhAPC in septic shock patients are currently underway [17,18]. We examined the association between rhAPC and outcomes in septic shock patients in our institution, with the hypothesis that patients with septic shock who were treated with rhAPC had an improved in-hospital mortality compared to patients with septic shock with similar acuity who did not receive rhAPC.

## Materials and methods

### Subjects

This investigation is a retrospective cohort study of patients admitted to a large university affiliated hospital with 56 medical-surgical intensive care unit (ICU) beds, between July 2003 and February 2009. Data were obtained from a large, multiinstitutional, critical care patient data set (Project Impact Critical Care Data System). Project IMPACT (PI) is a comprehensive database system developed to measure and describe the care of ICU patients. Developed by a multidisciplinary group of critical care expert members of the Society of Critical Care Medicine, this system allows practitioners to quantify practice patterns and patient outcomes and compare them with those of similar ICUs. Personnel at >100 ICUs at multiple participating hospitals enter information for >100 defined patient data elements into the PI software. Data elements include patient demographics, treatment, outcomes, complications, and resource utilization. On a quarterly basis, data from all hospitals are merged into the PI central database. Reports are then generated that include both summary and individual hospital data. Specifically, data on age, height, ICU admission weight, gender, comorbidities, organ failures, and ICU and hospital LOS were collected. PI data at our institution were collected by experienced registered ICU nurses (more than 16 years experience). Additional data elements were collected from the patients' electronic medical records.

Patients were included if they were 18 years of age or older, had a principal diagnosis of sepsis as defined by the 1992 SCCM/ACCP consensus conference definition [19], admitted to the ICU, and if they developed systolic blood pressure less than 90 mmHg, not responding to appropriate fluid resuscitation, and requiring vasopressors to maintain mean arterial pressure greater than or equal to 65 mmHG [19]. All sepsis patients in our institution are managed based on current sepsis management guidelines [20,21].

Baseline characteristics including demographic information and information about preexisting conditions, organ function/failure, infection, and pertinent medications were collected. APACHE II scores and sequential organ failure assessment (SOFA) scores were calculated. In all patients that received rhAPC, the infusion was started within 24 hours of development of septic shock. Presence or development of acute organ failures was documented. Organ failure definitions are shown in Table 1. The conduction of this study was approved by the Institutional Review Board at St. John's Mercy Medical Center (Approval #10-084). The board waived the need for informed consent.

**Table 1:** Definitions of organ failures

Organ	Definition
Cardiovascular	Blood pressure criteria are present for at least one hour despite adequate fluid resuscitation; arterial systolic BP <90 mmHg OR Arterial systolic BP with 40 mmHg drop from baseline OR mean arterial pressure (MAP) <70 mmHg, and vasopressors are required to maintain BP >90 mmHg systolic or MAP >70
Metabolic	Serum lactate elevated >upper limits per lab
Renal	Serum creatinine increased by more than 1 mg/dl from baseline after adequate fluid resuscitation OR serum creatinine > = 2 mg/dl, in the absence of known baseline *^a^*
Respiratory	PaO2/FiO2 < = 300 OR requires PEEP >5 cm H2O*^b^*
Hematologic	Platelet count is half the highest value in the last three days OR Platelets <100,000/mm^3 ^OR PT/PTT >1.5 times control*^c^*

*^a ^*Does not apply to patients on chronic hemodialysis.

*^b ^*Criteria apply to acute lung injury, not CHF or cardiogenic pulmonary edema.

*^c ^*Not due to anticoagulant therapy.

Abbreviations: BP, blood pressure; mmHG, millimeters of mercury; mg, milligram; dl, deciliter; PaO2, partial pressure of oxygen in arterial blood; FiO2, fraction of inspired oxygen; PEEP, positive end-expiratory pressure; PT, prothrombin time; PTT, partial thromboplastin time.

### Outcomes

The primary outcome measure was in-hospital mortality. We also recorded complications, including the occurrence of intracranial hemorrhage, gastrointestinal hemorrhage, major transfusions, and minor transfusions. Major transfusion was defined as the administration of six or more units of red blood cells within two consecutive days or four or more units in any single day. Minor transfusion was defined as the administration of two or three units of packed red blood cells in a single day. ICU-specific outcomes included length of stay, number of days spent on vasopressors and number of days on mechanical ventilation. We also assessed hospital length of stay.

### Analysis

Summary statistics were calculated for categorical and numeric data. For categorical data, frequencies and proportions (as percentages) are reported. For numeric data, means, standard deviations, medians and lower and upper quartiles are reported. Propensity for treatment with rhAPC was estimated by fitting a nonparsimonious logistic regression model containing 22 independent variables (Table 2). These variables were selected because they conceivably could bear on the choice to administer rhAPC. The c statistic was 0.75. The Hosmer-Lemeshow chi-square was 12.55 (df = 8), with a non-significant *P*-value of 0.13, which indicates the model is well-calibrated. Once the propensity scores were obtained, we used the recently described "fine balance" method of Rosenbaum, Ross, and Silber to produce 1:1 matched subjects [22]. Out of an overall number of 563 cases, we obtained 108 individuals treated with rhAPC matched to 108 control individuals not treated with rhAPC.

**Table 2:** Characteristics of patients with septic shock in the entire study cohort and propensity-matched sample

	Overall *n *= 563 n (%)	Treated matched *n *= 108 n (%)	Untreated matched *n *= 108 n (%)	*P ^a^*
**Patient characteristics**				
Age in yrs, mean, SD *^c^*	67.1(15.5)	62.9 (17.2)	64.2 (16.1)	0.53
Male *^c^*	284 (50.4)	60 (55.6)	57 (52.8)	0.64
Body mass index, mean, SD *^c^*	28.0 (8.6)	27.3 (7.8)	27.8 (7.5)	0.66
APACHE II, mean, SD *^c^*	24.8 (8.3)	24.5 (8.4)	23.9 (8.3)	0.54
				
**Comorbidities**				
Congestive heart failure *^c^*	86 (15.3)	10 (9.2)	15 (13.9)	0.28
Diabetes *^c^*	172 (30.5)	25 (23.2)	20 (18.5)	0.40
Hypertension *^c^*	320 (56.9)	51(47.2)	53 (49.1)	0.76
Chronic pulmonary disease *^c^*	266 (47.3)	42 (38.9)	40 (37.0)	0.77
Neurologic disease *^c^*	8 (1.4)	1(0.9)	0 (0)	1.00
Chronic kidney disease *^c^*	132 (23.5)	22 (20.4)	24 (22.2)	0.73
Cancer *^c^*	121(21.5)	7 (6.5)	7 (6.5)	1.00
Chronic liver disease *^c^*	36 (6.4)	10 (9.3)	8 (7.4)	0.62
Chronic pancreatitis *^c^*	3 (0.5)	1(0.9)	1(0.9)	1.00
				
**Organ failure**				
Kidney *^c^*	161(28.6)	33 (30.6)	34 (31.5)	0.88
Respiratory *^c^*	296 (52.6)	61 (56.5)	61 (56.5)	1.00
Hematologic *^c^*	214 (38.0)	52 (48.1)	49 (45.4)	0.67
Metabolic/lactic acidosis *^c^*	262 (46.5)	60 (55.6)	60 (55.6)	1.00
				
**Number of organ failures**				
1	104 (18.5)	13 (12.0)	12 (11.1)	
2	169 (30.0)	30 (27.8)	32 (29.6)	
3	147 (26.1)	30 (27.8)	32 (29.6)	0.97*^b^*
4	102 (18.1)	24 (22.2)	20 (18.5)	
5	41 (7.3)	11 (10.2)	12 (11.1)	
				
Inotrope *^c^*	123 (21.9)	30 (27.8)	32 (29.6)	0.77
Steroid *^c^*	219 (38.9)	50 (46.3)	46 (42.6)	0.59
Insulin drip *^c^*	153 (27.2)	37 (34.3)	32 (29.7)	0.46
Statins *^c^*	65 (11.6)	19 (17.6)	16 (14.8)	0.59
Chemical DVT prophylaxis *^c^*	319 (56.7)	76 (70.4)	74 (68.5)	0.77
				
**Sepsis source**				
Abdomen	86 (15.3)	12 (11.1)	20 (18.5)	
Blood	34 (6.0)	9 (8.3)	3 (2.8)	
Lung	251 (44.6)	54 (50.0)	42 (38.9)	0.08*^b^*
Urinary Tract	101 (17.9)	20 (18.5)	22 (20.4)	
Other/unknown	91 (16.2)	13 (12.0)	21 (19.4)	

*^a ^*The *P-*value for treated matched vs. untreated matched adjusted for matching by propensity.

*^b ^P*-values, one for the number of organ failures, and one for sepsis source, because each test is being done as a chi-square test on a 2 × 5 crosstable.

*^c ^*The 22 variables used in the propensity matching.

Abbreviations: APACHE, Acute Physiology and Chronic Health Evaluation; DVT, deep vein thrombosis; *P*, *P*-value; SD, standard deviation; yrs, years.

Table 2 shows the characteristics of the treated and untreated matched subjects. No statistically significant differences were found, indicating that the matching process succeeded in identifying comparable pairs of individuals. All statistical tests took into account the paired nature of the data. There may be some differences between the overall cohort and the matched groups, but propensity matching has proved to be the best method for dealing with those differences. Table 3 shows the outcomes by treatment. All statistical tests took pairing into account, except for comparisons of lengths of stay and days on ventilator and vasopressors. These four comparisons were limited to survivors, which caused pairing to be broken.

**Table 3:** Outcomes of patients with septic shock in the entire study cohort and propensity-matched sample

	**Overall**, *n *= 563 n (%)	**Treated matched**, *n *= 108 n (%)	**Untreated matched**, *n *= 108 n (%)	*P ^a^*
**Main outcomes**				
Length of stay, median days, (IQR)^b^	22 (12, 39)	19.5 (11, 33)	27 (12, 45)	0.11
Hospital mortality	266 (47.2)	38 (35.2)	57 (53.8)	0.005
**Intensive care unit outcomes**				
Mortality	226 (40.1)	36 (33.3)	47 (43.5)	0.09
Length of stay, median days, (IQR)	9.3 (4.0, 21.3)	11 (6, 22)	11.3 (4.5, 28)	0.90
Days on ventilator, median, (IQR)	8.8 (3.7, 22.6)	9 (6, 22)	8.7 (4.3, 24)	0.80
Days on vasopressors, median, (IQR)	2 (1, 3)	2 (1, 4)	2 (1, 3)	0.90
**Complications**				
Gastrointestinal bleeding	9 (1.6)	3 (2.8)	0 (0.0)	1.00
Intracranial bleeding	1 (0.2)	0 (0)	0 (0)	1.00
Minor transfusion	216 (38.4)	38 (35.2)	45 (41.7)	0.27
Major transfusion	45 (8.5)	4 (3.7)	10 (9.3)	0.09
Any transfusion(minor and/or major)	243 (43.2)	41 (38.0)	50 (46.3)	0.16

*^a ^*The *P-*value for treated matched vs. untreated matched adjusted for matching by propensity.

^b ^IQR, interquartile range.

## Results

A total of 563 septic shock patients were included (110 received rhAPC and 453 did not). Treated and untreated groups were matched in patient characteristics, comorbidities, physiologic variables, organ failures, and APACHE II scores (Table 2). Mean APACHE II scores were 24.5 for the matched treated and 23.9 for the matched untreated group (*P *= 0.54). In a 1:1 propensity-matched analysis, receipt of rhAPC was associated with reduced in-hospital mortality (35.2% vs. 53.8%, *P *= 0.005), similar mean days on vasopressors (2 vs. 2, *P *= 0.90), similar mean days on mechanical ventilation (9 vs. 8.7, *P *= 0.80), similar mean length of ICU stay in days (11.0 vs. 11.3, *P *= 0.9), and similar mean length of hospital stay in days (19.5 vs 27, *P *= 0.11). No patients in either group had intracranial bleeding. Differences in major transfusions (3.7% vs 9.3%, *P *= 0.09), minor transfusions (35.2% vs 41.7%, *P *= 0.27), any transfusion (minor and/or major, 38% vs 46.3%, *P *= 0.16) and gastrointestinal bleeding (2.8% vs 0%, *P *= 1.00) were not statistically significant (Table 3).

## Discussion

In this cohort propensity-matched analysis study, we found that patients with septic shock who were treated with rhAPC had a reduced in-hospital mortality compared with patients with septic shock with similar acuity who were not treated with rhAPC, with a relative risk reduction of 34.6%, and absolute risk reduction of 18.6%. This translates into a number needed to treat (NNT) to prevent a death of 5.4. We also found that the rate of bleeding in our cohort was similar to other published studies. In the major clinical trials, over the 28-day study period, serious bleeding events were observed in 3.5 to 6.5% of patients receiving rhAPC as compared with 2.0 to 5.0% of patients receiving the placebo [23]. Rates of intracranial hemorrhage were 0 to 1.5% with rhAPC as compared with 0 to 0.7% with the placebo.

The first large randomized trial studied a dose of 24 μg per kilogram of body weight per hour in the phase 3 PROWESS study [8]. This placebo-controlled, randomized, double-blind, multicenter trial included 1,690 patients. Treatment with rhAPC within 24 hours after diagnosis was associated with a mortality rate of 24.7% at 28 days versus 30.8% with placebo (*P *= 0.005). The overall incidence of at least one bleeding event was 24.9% in the treatment group and 17.7% in the placebo group (*P *= 0.001) [24]. The incidence of serious bleeding in the PROWESS study was also higher in the rhAPC group than in the placebo group (3.5% vs. 2.0%, *P *= 0.06). In subgroup analyses, most of the benefit of rhAPC treatment was seen in patients at increased risk for death, including those with APACHE II scores of 25 or greater. A follow-up study, the Administration of Drotrecogin Alfa (Activated) in Early Stage Severe Sepsis (ADDRESS) trial, evaluated the role of rhAPC in patients with severe sepsis, associated with either single-organ failure or an APACHE II score below 25 [10]. The study was stopped, after enrolling 2,640 patients, because there was no indication of a positive effect. The 28-day rate of death from any cause was 18.5% in the rhAPC group and 17% in the placebo group (*P *= 0.34). The in-hospital mortality rate was 20.6% in the rhAPC group and 20.5% in the placebo group (*P *= 0.98). Serious bleeding occurred in 2.4% of patients receiving rhAPC and in 1.2% of patients receiving the placebo (*P *= 0.02) during the drug-infusion period. No significant difference was observed in the rate of intracranial hemorrhage (0.3% with rhAPC and 0.2% with the placebo) [10].

The efficacy of rhAPC in severe sepsis remains a matter of dispute. Some experts even criticized the Food and Drug Administration's decision to approve rhAPC [24-26]. The main criticisms were aimed at intercurrent changes made in the PROWESS protocol, among them the exclusion of participants who were thought to be likely to die within 28 days because of the severity of the underlying disease and the introduction of new cell lines for growing the rhAPC protein. The use of a subgroup analysis of data from a major trial to identify suitable candidates for treatment was also heavily criticized. Mackenzie, Carlet and others suggested that a general recommendation for the clinical use of rhAPC was premature or not justified [27,28]. On the other hand, Surviving Sepsis Guidelines suggested the use of rhAPC for severe septic patients who have multiorgan failure or a high risk of death [21]. As a result, two large multicenter, randomized, double-blind trials investigating the use of rhAPC in septic shock are currently underway [17,18].

The patients in our trial had mortality rates higher than those observed in the PROWESS trial, possibly because we only studied septic shock patients that might be sicker than the patient population in PROWESS. In our study, we had fewer patients with single organ failure, a similar number of patients with two or three organ failures, but more patients with four or five organ failures than PROWESS. In our study, the more organ failures, the higher was the mortality in both groups. Across all organ failure subgroups (one, two, three, four, or five organ failure subgroups), in-hospital mortality was persistently higher in the group that did not receive rhAPC than the group that received rhAPC (data not shown). The higher relative risk reduction in mortality in our study (33.9% vs 13% in PROWESS) is likely due to the fact that rhAPC is most beneficial in the sickest septic shock patients, with multiple organ failures. The bleeding rate in our study was smaller than that observed in PROWESS, but similar to that observed in other major clinical trials [23].

In our study, there was no difference between the matched treated and matched untreated groups in days on vasopressors, days on mechanical ventilation, length of ICU stay in days, and length of hospital stay in days (Table 3). These findings are not surprising and are similar to those found in PROWESS [9]. This adds to the evidence that survivors of treated versus untreated groups look fairly similar, but more patients survived in the treated group. This also suggests that rhAPC improves hospital survival without the consumption of additional resources.

Our study is limited in that it is a retrospective analysis and that the number of patients is relatively small. However, this propensity analysis provides an estimate of the benefit of rhAPC in a natural hospital setting. That makes it different from an estimate made from a randomized controlled trial (RCT), where conditions are more controlled. An RCT tells us that there is a causal effect of a certain size. A propensity analysis of retrospective data tells us what size effect we can expect under normal hospital conditions. Also, fewer patients (110) received rhAPC than those who did not (453). This could be explained by the possibility that treatment was preferentially directed toward patients thought to have a higher likelihood of meaningful survival, a lower risk of bleeding, or contraindication to rhAPC by the managing intensivist. However, it is not explained by differences in the types of patients receiving it versus not since we used all possible recorded pre-treatment variables. In addition, the 108 untreated patients are also candidates for rhAPC therapy in the sense that they have propensities to receive rhAPC that match the propensities of those 108 patients actually receiving rhAPC. There was also no evidence of a difference in any of the bleeding variables between the two groups (Table 3). Usage of rhAPC also depended upon whether the intensivist caring for the patient was critical or supportive of rhAPC. However, usage of rhAPC has remained constant over the study period, seen graphically and by non-significant hypothesis tests (t-test, Wilcoxon test and logistic regression all have *P*-values near 0.5). Our primary outcome measure was in-hospital mortality; we did not have access to longer-term outcomes, and, thus, our survival analyses assumed patients who were discharged alive from the hospital were in fact alive at Day 28. However, this study is made stronger by the propensity-matched analysis. Our primary outcome was in-hospital mortality, and rhAPC began to show its effect in the ICU (Table 3). ICU mortality comes close to statistical significance with *P *= 0.09; however, if we make the test one-sided in the hypothesized direction, the *P*-value becomes 0.045. In-hospital mortality shows fuller statistical significance (which of course includes ICU mortality). A recent similar study conducted by Lindenauer *et al*. showed similar results [29]. In a propensity-matched sample in which all covariates achieved balance, receipt of rhAPC was associated with reduced hospital mortality (40.7% vs. 46.6%; risk ratio, 0.87; 95% confidence interval, 0.80 to 0.95). Four rhAPC-treated patients (0.25%) had hemorrhagic stroke, 107 (6.8%) had gastrointestinal bleeding, and five (0.3%) required major transfusion [29]. An important limitation of the study by Lindenauer *et al*. is that all the data on their patients were based on billing records, and not direct clinical measurements.

## Conclusions

Patients with septic shock who were treated with rhAPC had a reduced in-hospital mortality compared with patients with septic shock with similar acuity who were not treated with rhAPC. In addition, time on mechanical ventilation, time on vasopressors, length of ICU stay and bleeding complications did not differ between the groups. Pending results from large multicenter studies evaluating rhAPC in septic shock patients, our analysis adds to the evidence that early initiation of rhAPC may improve survival of patients with septic shock who are at low risk of bleeding.

## Key messages

• The efficacy of recombinant human activated protein C (rhAPC) in sepsis remains a matter of dispute.

• Pending results from two large multicenter, randomized, double-blind trials, we examined the association between treatment with rhAPC and outcomes among patients with septic shock in routine clinical practice in a large university-affiliated Intensive Care Unit.

• We performed a propensity-matched analysis, which equalizes the probability of receiving rhAPC, mimicking a randomized comparison.

• Patients with septic shock who were treated with rhAPC had reduced in-hospital mortality compared with patients with septic shock with similar acuity who were not treated with rhAPC, with no difference in complications.

• Our analysis provides an estimate of the benefit of rhAPC in septic shock patients in a natural hospital setting.

## Abbreviations

ADDRESS: The Administration of Drotrecogin Alfa (Activated) in Early Stage Severe Sepsis; APACHE II: Acute Physiology And Chronic Health Evaluation II; ICU: Intensive Care Unit; NNT: number needed to treat; PI: Project IMPACT; PROWESS: Prospective Recombinant Human Activated Protein C Worldwide Evaluation in Severe Sepsis; RCT: randomized controlled trial; rhAPC: recombinant human activated protein C; SOFA: sequential organ failure assessment.

## Competing interests

The authors declare that they have no competing interests.

## Authors' contributions

FS contributed to conceiving the study, acquiring and managing the data, analyzing the data and interpreting the results, drafting and revising the manuscript, and approving the manuscript in its final form. JO, MM, JS and AV contributed to acquiring and managing the data, revising the manuscript, and approving the manuscript in its final form. RT contributed to analyzing the data and interpreting the results, revising the manuscript, and approving the manuscript in its final form.
